# Impact of a multifaceted intervention to improve the clinical management of osteoporosis. The ESOSVAL-F study

**DOI:** 10.1186/1472-6963-10-292

**Published:** 2010-10-21

**Authors:** José Sanfélix-Genovés, Salvador Peiró, Gabriel Sanfélix-Gimeno, Isabel Hurtado, Manuel Pascual de la Torre, José Luis Trillo-Mata, Vicente Giner-Ruiz

**Affiliations:** 1 Centro Superior de Investigación en Salud Pública (CSISP), Valencia, Spain; 2 Centro de Salud de Nazaret, Departamento de Salud 5, Agencia Valenciana de Salud, Valencia, Spain; 3 Oficina de Abucasis, Agencia Valenciana de Salud, Valencia, Spain; 4 Dirección General de Farmacia y Productos Sanitarios, Agencia Valenciana de Salud, Valencia, Spain; 5 Centro de Salud Ciudad Jardín, Departamento de Salud 19, Agencia Valenciana de la Salud, Alicante, Spain

## Abstract

**Background:**

A study to evaluate the impact of a combined intervention (in-class and on-line training courses, a practicum and economic incentives) to improve anti-osteoporosis treatment and to improve recordkeeping for specific information about osteoporosis.

**Methods/design:**

A before/after study with a non-equivalent control group to evaluate the impact of the interventions associated with participation in the ESOSVAL-R cohort study (intervention group) compared to a group receiving no intervention (control group). The units of analysis are medical practices identified by a Healthcare Position Code (HPC) referring to a specific medical position in primary care general medicine in a Healthcare Department of the Region of Valencia, Spain. The subjects of the study are the 400 participating "practices" (population assigned to health care professionals, doctors and/or nurses) selected by the Healthcare Departments of the Valencia Healthcare Agency for participation as associate researchers in the ESOSVAL-R study (intervention group), compared to 400 participating "practices" assigned to primary care professionals NOT selected for participation as associate researchers in the ESOSVAL-R study, who are selected on the basis of their working in the same Healthcare Centers as the practices receiving the interventions (control group). The study's primary endpoint is the appropriateness of treatment according by the Spanish National Health System guide (2010) and the National Osteoporosis Foundation (NOF, 2008) and International Osteoporosis Foundation guidance (IOF, 2008).

The study will also evaluate a series of secondary and tertiary endpoints. The former are the suitability of treatment and evaluation of the risk of fracture; and the latter are the volume of information registered in the electronic clinical records, and the evaluation of risks and the suitability of treatment.

## Background

Osteoporosis is a systemic disease of the skeleton characterized by a loss in the bone's resistance increasing the risk of fracture. Resistance decreases when the bone's micro- and macro-structures deteriorate and bone mass is lost [1]. Osteoporosis is highly prevalent (1 in 3 women over the age of 50 in the Spanish setting [2]) with an incidence that is rising as a result of the greater life expectancy enjoyed by many of today's societies. While osteoporosis is a silent disease, it has a very important clinical impact because it involves a major risk of bone fracture. The significant events associated with osteoporosis are osteoporotic fractures, and their associated morbidity and mortality. Osteoporotic fractures most frequently occur in the thoracolumbar spine, the hip and the wrist. Although the risk of fracture increases in individuals with low bone mass, a previous osteoporotic fracture is the best predictor for new vertebral and hip fractures [3,4].

Osteoporotic fractures have important social and healthcare repercussions. Mortality due to hip fracture in hospitalized patients is between 5 and 8% and jumps to 20-30% during the first year [5]; only one third of hip fracture survivors recover their pre-fracture condition. The presence and number of vertebral fractures has also been related to a loss in quality of life [6,7] and greater long-term mortality [8,9]. Vertebrae fractures, the most frequent of osteoporotic fractures, are both under-diagnosed and under-treated [2,10,11]. A wide range of drugs are used to reduce the risk of osteoporotic fracture, but there are no broadly accepted criteria to estimate risk or to select patients for treatment. Internationally, one of the priorities of clinical research in the field is to develop a set of simple clinical criteria that can be used in daily practice to help doctors decide whom to treat, and when to initiate treatment.

The Valencia Health Agency, the public healthcare network providing healthcare close to 95% of the inhabitants of the Region of Valencia, Spain, has been implementing in the last years an electronic clinical record system, called ABUCASIS. For practical purposes, ABUCASIS becomes a population-based electronic database enabling the monitoring of the most relevant clinical and therapeutic data and allowing longitudinal studies on the 5 million people attending the Valencia Health Agency healthcare centres.

The Valencia osteoporosis study ESOSVAL [12] is one of the cornerstones of the 2nd Plan for the Prevention and Follow-Up on Osteoporosis in the Region of Valencia that has been initiated by the Regional Ministry of Health [13]. Its aims are to improve osteoporosis care and reduce the risk of fracture by providing clinical training for professionals, modifying the ABUCASIS clinical records system with specific changes related to osteoporosis prevention and management. The Plan will also promote research that can be applied in routine clinical practice. The ESOSVAL project involves the training of 800 primary care doctors and nurses who participate in the ESOSVAL-Risk (ESOSVAL-R) study [12]. These professionals will use the modified ABUCASIS computerized clinical records to follow-up 14,500 patients during 7-10 years. The main objective of the ESOSVAL-R project is to develop a scale to predict the risk of osteoporotic fracture in any location, and in the hip, to serve as the base to establish criteria for the efficient treatment of osteoporosis [12].

The professionals who participate in the ESOSVAL-R project will engage in a multifaceted intervention - the ESOSVAL Formation (ESOSVAL-F) study - that includes training, practical activities and some economic incentives. At the start of the project, the participants receive four hours of practical training on collecting and recording information about osteoporosis in ABUCASIS. Then they recruit and follow-up on the patients who will take part in the ESOSVAL-R study. This involves collecting information relevant to the study from the clinical records of 18 participating patients under their care. Participation in the study has, furthermore, been included as an "indicator" to obtain points towards the financial incentives included in the Valencia Health Agency's Management Contract. Additionally, all participating professional are engaged in an on-line training course on osteoporosis prepared by recognized experts in the field. The intervention will conclude with further classroom reinforcement in the first quarter of 2011.

The main objective of the ESOSVAL-F study is to evaluate the impact of this multifaceted intervention on improvements in the appropriateness of anti-osteoporosis treatment, and improvements in recordkeeping of the information that is specifically related to osteoporosis and osteoporotic fractures in the ABUCASIS electronic clinical records, comparing the results of the medical professionals participating in the ESOSVAL-R study (Intervention Group) with those clinicians who are not participating in the project (Control Group).

## Methods/Design

### Main Objective

To evaluate the impact of a multifaceted intervention on the improvement of the appropriateness of anti-osteoporosis treatment and on the inclusion of specifically related to osteoporosis information in the electronic clinical records.

### Specific objectives

1. To describe the appropriateness of treatments and the volume of specific information related to osteoporosis at the beginning of intervention (baseline situation).

2. To analyze any possible baseline differences in managing osteoporosis between the Intervention and the control group.

3. To evaluate the impact of the multifaceted intervention, taking into account any differences between control and intervention groups in appropriateness of treatment and in the quality and volume of the information on osteoporosis registered once the intervention has finished.

4. To analyze the degree of improvement in the appropriateness of treatment and in the thoroughness of recordkeeping throughout the intervention period, separately for both groups, and analyze any differences in these changes by comparing the values collected after the interventions with the baseline values for both groups.

### Design

Before/after study with a non-equivalent control group to evaluate the impact of the interventions associated with participation in the ESOSVAL-Risk cohort study.

To summarize very briefly, the ESOSVAL-R study is being conducted with aprox. 14,500 men and women recruited from all over the Region of Valencia into 400 practices under the care of participating primary care doctors and nurses [12]. These persons will be followed for 7 to 10 years to evaluate various objectives related to the incidence of osteoporotic fractures. To facilitate follow-up of these patients, the ESOVAL-R project implemented various changes to the system used to register information in the patients' clinical records throughout the entirety of the Valencia Healthcare Agency, and will conduct training activities with the participating healthcare professionals, some of which are associated with incentives of various type. The impact of these interventions is what is being evaluated in the current ESOSVAL-F project.

### Setting

Region of Valencia, Spain, and, specifically, the primary care network depending from the Valencia Health Agency. In the Region of Valencia, as in Spanish National Health System, coverage is practically universal, with 97% of the population served by the Valencia HealthCare Agency administered by the Regional Government. The Agency is structured in 24 main Health Districts (where a geographical area with about 200,000 people is served by one public hospital), and 234 geographically delimited Basic Health Areas, with 5,000-25,000 people served by a Primary Healthcare Centre. Other noteworthy features of the Valencia HealthCare System are the cost-free status of care, hospital and primary care funding by means of government budgets and the fact that doctors, who enjoy a civil servant-like status, are paid by salary with discrete economic incentives linked to performance in a pool of organizational and clinical indicators defined annually in the so-called "Health Agency's Management Contract".

### Population and sample

The units of analysis of the ESOSVAL-F are the medical practices (the people registered with each general practice doctor) of the Healthcare Departments of the Region of Valencia. The intervention is directed to the doctors and/or nurses attending these practices. Inclusion criteria were the following:

- Intervention group: was integrated by Medical Practices participating in the ESOSVAL-R study. To be selected the corresponding professional should occupy a titular position as general practice doctor o nurse (excluding out-of-hours, temporal substitutes and emergency professionals) with anticipated continuity at least three months after ESOSVAL-R recruitment, have an operating email address and a computer with internet access, and agree for participation in the study.

- Control Group was integrated for medical practices NOT selected for participation in the ESOSVAL-R study. These practices were selected on the basis of their belonging to one practice of the intervention group. If possible, we choose the same Healthcare Centre and a practice with similar timetable and size of registered patients. When this was not possible, another one belonging to the same Department was selected.

The size of the sample in the Intervention Group (n = 400 general practices) was predetermined by the objectives of the ESOSVAL-R study [12]. The same number of practices was recruited for the Control Group (n = 400). Assuming a type I error of 0.05 (two tailed-tests) and a power of 0.90, 400 units in each branch of the study (ratio = 1 between groups) provides sufficient power to be able to detect differences in the study's main variable.

### Intervention

Given the characteristics of the ESOSVAL project linked to the Regional's Plan of Osteoporosis, both the Intervention and the Control Group will receive some form of intervention aimed at improving care. The Control Group will benefit from the improvements introduced by the ESOSVAL project in the ABUCASIS Electronic Clinical Records system, since they affect all the system's users, the doctors and nurses providing healthcare, including those in the Control Groups. These improvements consist in the incorporation of a new follow-up sheet for patients with osteoporosis or risk factors for osteoporosis, and a series of tables, scales and variables that can be monitored to improve the care and follow-up of these patients. The implementation of this change in the patients' clinical records will be done through the usual training process used by the Valencia Healthcare Agency to introduce any change in recordkeeping (an informational session, and the option to have any individual questions answered).

The intervention group, and apart from the above mentioned changes to the recordkeeping system, receive a multifaceted intervention: 1) The participating clinicians took a four-hour classroom course in the last quarter of 2009, held in each Department; 2) Next, they participated in recruiting and following-up on patients for the ESOSVAL-R study. This requires the healthcare providers to include relevant information in the clinical records of 18 patients, and involves a hands-on practicum in obtaining information about osteoporosis and its incorporation into the clinical records; 3) participation in the study has been included as an "indicator" towards gaining points in the Valencia Health Agency's Management Contract, that will lead to economic incentives; 4) An on-line course on osteoporosis will be given during the first, third and fourth quarters of 2010. It is organized in modules prepared by recognized national experts; 5) During the first quarter of 2011, after all the participating healthcare providers have completed the on-line course, another classroom course will be given to reinforce training and to divulge the results collected so far during the intervention (Table 1). The courses will be given to the doctors and nurses in the Region's Healthcare Departments who volunteer for participation in the project and who work with the medical practices selected.

**Table 1:** ESOSVAL-Formation Project chronogram

Year	2009				2010				2011	
Quarter	1	2	3	4	1	2	3	4	1	2
Design of the Training Plan	►	►	►▐							

Design of the Research Project: ESOSVAL-F			►▐							

Presentation to Ethical Committee	►▐									

Changes to the electronic clinical history system to improve recordkeeping on osteoporosis	►	►	►▐							

In-class training courses in the Healthcare Departments				►▐				►▐		

"On-line" training					►	►		►▐		

Statistical analysis									►	►▐

Issue of report on results										►►

### Outcomes

The expected short-term results of the intervention are to improve the recordkeeping of information about osteoporosis and osteoporotic fracture and to improve appropriateness of treatment and the request of diagnostic tests. The expected long term results of the intervention are to reduce the incidence of osteoporotic fracture. The evaluation currently being conducted in this study intends to examine only the expected short term results. The evaluation of the long-term results will require from seven to ten years of follow-up. In this context, results will be measured as:

### Main endpoint

Appropriateness of treatment according by the Spanish National Health System guide (2010) [14] and the National Osteoporosis Foundation (NOF, 2008) [15] and International Osteoporosis Foundation guidance (IOF, 2008) [16].

### Secondary endpoints

1) Percentage of people ≥70 years diagnosed with osteoporosis and/or osteoporotic fracture treated with alendronate, risendronate, ibandronate or strontium ranelate, compared to all people ≥70 years in the practice. These values will also be analyzed with a breakdown for gender; 2) Percentage of patients ≥50 years NOT diagnosed with osteoporosis nor osteoporotic fracture treated with anti-osteoporotic drugs, compared to the total population ≥50 years in the practice, total and by gender; 3) Percentage of patients ≥50 and ≤ 65 years with a diagnosis of osteoporosis/osteopenia without osteoporotic fracture, receiving treatment with anti-osteoporotic drugs; 4) Percentage of patients ≥50 years in treatment with calcium and/or vitamin D, with a diagnosis of osteoporosis and/or osteoporotic fracture; 5) Percentage of patients ≥70 years in treatment with raloxifene, teriparatide, PTH or calcitonin, with a diagnosis of osteoporosis and/or osteoporotic fracture; 6) Percentage of patients ≥70 years in treatment with raloxifene, teriparatide, PTH or calcitonin, with no diagnosis of osteoporosis or osteoporotic fracture. 7) Percentage of patients ≥50 diagnosed of osteoporotic fracture (vertebra, wrist, proximal humerus or other specific and non-specific sites), compared to the total population of patients ≥50 years in the practice, total, by type of fracture, age group and gender; 8) Percentage of patients ≥50 years who diagnosed of osteoporosis/osteopenia (OMS criteria), compared to the total population of patients ≥50 years in the practice, total, by age group and by gender.

### Tertiary endpoints

1) Percentage of patients ≥70 years for whom the "evaluation of the risk of fall" form has been filled in, compared to the total population ≥70 years in the respective practices, total and by gender; 2) Percentage of patients ≥50 years with a diagnosis of a sedentary life-style, compared to the total population of patients ≥50 year in the practice, total and by gender; 3) Percentage of women ≥50 years with a diagnosis of early menopause, compared to the total women of patients ≥50 years in the practice; 4) Percentage of patients ≥50 years for whom the "Evaluation for family history of osteoporotic fractures" form has been filled in, compared to the total population of patients ≥ 50 years in the practice; 5) Percentage of patients ≥ 50 years for whom the "Evaluation for calcium intake" form has been filled in; 6) Percentage of patients ≥50 years for whom the evaluation for smoking has been filled in; 7) Percentage of patients ≥50 years for whom the evaluation for educational level has been filled in; 8) Percentage of patients ≥ 50 years for whom the evaluation of body mass index has been filled in.

### Others variables and definitions

#### Associated with the practice

Healthcare Center, Basic Healthcare Zone, Health Department, timetable (for appointments with patients), size (number of patients assigned to the professional).

#### Associated with the doctor in charge of the practice

Age, sex, training (whether she/he had their Family Physician certificate after an nationally accredited 3-4 years residency program), contractual conditions, number of years practicing in primary care, number of years occupying the current position.

#### Associated with the patients in each practice

age, sex, body mass index, smoker status, drinker status, physical activity habits, antecedents of first degree family member with hip fracture, calcium intake, non-treated hypogonadism, rheumatoid arthritis, other diseases affecting the bones excluding hypogonadism, use of oral glucocorticoids, use of other drugs affecting the bones (excluding glucocorticoids), previous osteoporotic fracture, risk of fall, prolonged immobilization, osteoporosis of the lumbar spine assessed by DXA (T score for L2-L4), osteoporosis of the hip assessed by DXA for the whole hip or femur neck (T score), anti-osteoporotic treatment.

### Data sources

All of the data pertaining to the patients in the medical practice will be taken from the ABUCASIS Electronic Clinical Records system. The data concerning the practice and the doctors and nurses will be supplied by the Valencia Healthcare Agency.

### Development of the study

The study will be undertaken in accordance with usual conditions of clinical practice and good clinical practice. As per the working plan detailed below, changes in the clinical records come into effect in the computer system during the last quarter of 2009, simultaneously with the in-class training courses. Further training will be provided during 2010, and the information collected during the first quarter of 2011 will be used to compare the results of the Intervention and Control Groups.

### Statistical Analysis

1) Once the patients in all of the practices have been recruited, and at the beginning of the training period, the baseline information for both the IG and the CG will be analyzed. Information about the demographic features of the practices receiving healthcare and about the various results measured will be recorded, using the necessary parameters for each variable (means, proportions) with their corresponding 95% confidence intervals (CI95%). Additionally, any differences between the two groups will be analyzed using appropriate tests to measure differences in proportions. 2) One year after the start of the study, and coinciding with the completion of the on-line training, the parameters used in the baseline study will be re-evaluated to detect any differences in results between the IG and the CG. Additionally, paired tests will be used to analyze any before/after differences in the IG and the CG, and any improvements made in the groups will be compared with tests of differences between proportions. Controls will be used for the self-correlation expected between the results of patients under a single doctor's care. All of the statistical analyses will be done with the STATA program.

### Ethical aspects

The study will be conducted according to the international standards for epidemiological studies, as established in the International Guideline for Ethical Review of Epidemiological Studies (Council for the International Organizations of Medical Sciences, Geneva, 1991) and by the Spanish Society of Epidemiology on the review of the ethical aspects of epidemiological research. The study has been approved by the Committee for Ethics and Clinical Essays of the *Centro Superior de Investigación en Salud Pública *(Center for Public Health Research).

The ESOSVAL-F study is being conducted under the usual conditions of clinical practice; beyond the training interventions and those designed to help healthcare professionals improve their clinical practice, the study does not provide the participating patients with intervention in any form that is different from what is provided to non-participating patients. The participation of the doctors and nurses in the ESOSVAL-F study does not involve providing the patients with preventive procedures, or additional diagnostic or therapeutic measures, beyond what their doctors and nurses deem appropriate. Therefore, the participation of the doctors and nurses in the study does not include any added risk for the patients receiving their care. All of the interventions directed towards improving healthcare are part of improvement strategies being deployed throughout the healthcare system in its entirety (changes in the Clinical Records), or represent training interventions provided exclusively for healthcare staff (doctors and nurses).

The treatment of personal information in the ESOSVAL-F study complies with Spanish Organic Law 15/1999 and Law 41/2002 of November 14, the instruments which regulate patient autonomy and rights and obligations concerning clinical information and documentation. Article 16.3 of Law 42/2000 establishes that "access to clinical records for legal, epidemiological, public health, research or teaching purposes requires separating all data identifying the patients from all medical or health-related data, in such a way that, as a general rule, the patient's anonymity is preserved, unless the patient himself has given consent to not separating these [types of] data." All patient data obtained from the ABUCASIS system will be held anonymously and separately, and will solely be linked to a key that will exclusively be used in the context of the system, in such a way that only authorized personnel from the ABUCASIS office (and never members of the research team) will be able to associate such data to an identified or identifiable person. In no circumstances will members of the research team have access to data identifying the patients.

## Discussion

A series of limitations are foreseen in connection with this study:

### Selection bias

While criteria for inclusion into the intervention group include having volunteered for participation in the ESOSVAL-R study, the control group is composed for professionals who have not made such a request. This aspect suggests there may be differences in the baseline characteristics of the two groups, maybe with influence in the study endpoints. Strategies to minimize this risk include selecting for participation in both groups professionals from the same Healthcare Centers and with similar patient assignations. Nevertheless, as occurs in all studies with non-equivalent control groups, the selection bias cannot be entirely discarded.

### Information bias

These may occur when a clinical record is missing, or when information is not registered uniformly in the Electronic Clinical Record. The main problem here is that measuring the appropriateness of treatment and the quality of the information recorded about the patients depends on the amount of information registered in the system, and this volume may differ between the control and the intervention group. Although the volume of information itself is being used as measurable endpoints, the implications of "information not registered" are different from those of "inappropriate treatment". In order to limit this problem, simple indicators derived from data that is usually registered (treatment, patient age) have been defined as primary and secondary endpoints, rather than using more sophisticated ones that are highly dependent on the quality of the register.

### Maduration

The endpoints examined in the study are expected to improve over time for various reasons: The improvements made to the ABUCASIS electronic clinical record system should bear fruit; the study has an open design; and the participating doctors will experience a learning curve throughout the study. This bias will be limited by the use of a Control Group, and by evaluating the differences between both groupos before evaluating any differences observed before and after the interventions.

### Surrogate endpoints

The short-term design of the evaluation will not make it possible to assess the impact of the intervention on the most relevant clinical endpoint (the reduction of osteoporotic fractures), but will be limited to the surrogate endpoints, assuming that if the quality of the intermediate endpoints improves, the clinical endpoints will improve as well. Although this is not a limitation of the current study, which has been designed to measure short-term effects, the ESOSVAL Group will design an evaluation at five years to measure the long-term impact of the intervention.

## Abbreviations

ESOSVAL-R: Esosval Risk study; ESOSVAL-F: Esosval Training study; CSISP: Centro Superior de Investigación en Salud Pública; DXA: dual energy x-ray absorptiometry; NOF: National Osteoporosis Foundation; IOF: International Osteoporosis Foundation.

## Competing interests

The authors declare that they have no competing interests.

## Authors' contributions

JSG, SP and GSG carry out the design of the study and contributed with intellectual input in the design of this paper. JSG, IH, MPT, JLTM and VG contributed in several parts of the ESOSVAL-F Study (ABUCASIS modifications, database designs, tuition of participating clinicians). All authors contributed to the writing of the manuscript, corrected draft versions and approved the final manuscript.

## Pre-publication history

The pre-publication history for this paper can be accessed here:

http://www.biomedcentral.com/1472-6963/10/292/prepub
